# Dietary patterns and birth outcomes of healthy Lebanese pregnant women

**DOI:** 10.3389/fnut.2022.977288

**Published:** 2022-09-27

**Authors:** Tatiana Papazian, Pascale Salameh, Georges Abi Tayeh, Assaad Kesrouani, Carla Aoun, Mia Abou Diwan, Lydia Rabbaa Khabbaz

**Affiliations:** ^1^Department of Nutrition, Faculty of Pharmacy, Saint-Joseph University of Beirut, Beirut, Lebanon; ^2^Laboratoire de Pharmacologie, Pharmacie Clinique et Contrôle de Qualité des Médicaments, Faculty of Pharmacy, Saint-Joseph University of Beirut, Beirut, Lebanon; ^3^School of Medicine, Lebanese American University, Byblos, Lebanon; ^4^Institut National de Santé Publique, Épidémiologie Clinique et Toxicologie-Liban (INSPECT-LB), Beirut, Lebanon; ^5^Department of Primary Care and Population Health, University of Nicosia Medical School, Nicosia, Cyprus; ^6^Faculty of Pharmacy, Lebanese University, Hadat, Lebanon; ^7^Department of Gynecology and Obstetrics, Hotel Dieu de France Hospital, Beirut, Lebanon

**Keywords:** dietary patterns, pregnancy, neonatal outcomes, body mass index, gestational weight gain

## Abstract

**Background:**

The aim of this study was to define the dietary patterns (DPs) of a sample of Lebanese pregnant women and to establish their correlation with maternal and neonatal outcomes.

**Methods:**

A cross-sectional study was conducted among 358 Lebanese pregnant women. Maternal socio-demographic variables, anthropometric measurements, gestational weight gain and neonatal outcomes such as weight, length, head circumference and Apgar score were collected by qualified dietitians. Dietary intake was assessed by a validated food frequency questionnaire and three 24-h dietary recalls. DPs were determined, *a posteriori*, by a factor analysis to distinguish the inter-correlations between the food groups and a cluster analysis method to assemble the participants into groupings based on similarities in food consumption.

**Results:**

The identified DPs were not exclusively composed of specific food groups, since some components were overlapping in the DPs. The first one was characterized by a high consumption of starchy vegetables, unsaturated fats and unhealthy foods, the second was rich in fruits and vegetables, seeds, rice and pasta, and in fried local meals and the third was mainly based on protein-rich foods like poultry, fish, eggs and dairy products. They were named respectively as “Westernized,” “Mixed” and “Neo-Mediterranean” by the research team. Women having the lowest pre-gestational body mass index (BMI) and higher gestational age followed mainly the “Neo-Mediterranean” eating pattern.

**Conclusion:**

The three identified DPs among Lebanese pregnant women were correlated with the pre-gestational BMI, and some maternal variables. However, neither the maternal nor the neonatal outcomes were correlated with the DP adopted by the mothers.

## Background

Eating habits are the outcome of complex interactions of internal (hormonal, metabolic, physiological) and external (cultural, socio-demographic, economical) stimuli, leading the individual to select a specific set of certain foods, determining his dietary pattern (DP). Environmental and genetic factors affect the health of individuals, thus assessing single nutrient intake alone cannot accurately predict its impact on health and disease (1). Hence, the current approach in research is to move from the analysis of nutrients to a more holistic approach through the determination of DPs, in order to capture actual trends in dietary habits and achieve a reliable and valid dietary output, since people eat a combination of foods, not nutrients, composed of bioactive components that act synergically (2, 3).

Optimal fetal development and maternal health depend on a constant supply of high quality nutrients. In fact, higher adherence to healthier DPs was positively associated with better neonatal outcomes: appropriate birth weight, reduced fetal growth restriction and lower risk of preterm birth (2). Medical health authorities such as the World Health Organization (WHO) advise women to adopt a healthier lifestyle, monitor gestational weight gain, promote daily physical activity and abstain from smoking, alcohol and illicit drugs (2, 4, 5). Assessing the nutritional status of pregnant women and its short and long term repercussions on gestational complications such as diabetes (6), preeclampsia and adverse neonatal outcomes (7) is influenced by eating habits, environmental factors, and maternal hormonal fluctuations. The variability in research results justifies the need to conduct culture-based studies.

In a recent review and metanalysis published in 2019, adopting a “healthy” DP by pregnant women reduced the risk of preterm delivery and, hence improved several birth outcomes such as birth weight and gestational age (2).

Although future mothers become highly motivated and tend to adopt healthier eating habits, however, nutrition transition affects the eating patterns of all subgroups of the population worldwide. Lebanon is a small middle-income country on the Eastern shore of the Mediterranean Sea, reputed for its cuisine. However, during the last decades, the traditional Mediterranean meals have gone from high-fiber foods, such as non-refined cereals, nuts and seeds, fruits and vegetables, olive oil and vegetable-based proteins, to high-fat, low-fiber foods, rich in animal-based proteins and saturated fats (8). This dietary shift, together with the decline of the economic situation in the country, affected the nutritional status of the population, leading them to buy cheaper and unhealthy food alternatives, thus, increasing the prevalence of chronic diseases such as metabolic syndrome and cancer (9).

We hypothesized that pregnant women opting for an unhealthy DP, rich in refined foods, sugars and saturated fat would have a higher gestational weight gain and may encounter adverse maternal and neonatal outcomes. Because of the scarcity of the scientific publications related to the DPs of pregnant females living in the Mediterranean region, the aim of this research was primarily to identify the DPs of Lebanese pregnant females, and secondly to examine their associations with maternal factors, neonatal parameters and dietary intakes.

## Methods and materials

### Study design and population

This research is part of a prospective cohort conducted from 2014 till 2018, among pregnant Lebanese women, assessing the relationship between maternal dietary habits, inflammatory markers, genetic polymorphisms, together with gestational and neonatal outcomes. Healthy, non-vegan, Lebanese singleton pregnant females, aged between 18 and 40 years old, were recruited during their first prenatal consultation, post pregnancy confirmation, in private clinics in different geographic districts in the capital Beirut, in the regions of Mount Lebanon, and in the North and South. Field work was conducted between October 2016 and March 2018 by trained dietitians. The study protocol was approved by the Institutional Review Board of Saint-Joseph University (USJ) at Beirut Lebanon, the Hotel-Dieu Hospital Ethics Committee (CE HDF624/FP49) and the participating gynecologists. All participants gave their written consent prior to their participation for which no financial incentive was proposed.

### Maternal and neonatal data extraction

The research team, led by dietitians, conducted face to face interviews with the participants, to collect basic sociodemographic data and food recalls. Anthropometric measurements (weight and height) and age were taken from the medical files, or measured by a digital scale and a stadiometer, if data were unavailable. Body mass index (BMI) was calculated as the ratio of pre-gestational weight in kilograms to the square of height in meter. It was then categorized according to the WHO cut-off points (underweight < 18.5, normal 18.5–24.9, overweight 25–29.9 and obese >30) (10). The indicator of socio-economic status was the crowding index, calculated as the ratio of total co-residents per household, excluding newborn infants, on the number of rooms, excluding the kitchen and bathrooms. A crowding index more than one suggests a household with limited economic resources.

Throughout pregnancy, a follow-up was maintained with all participants *via* face-to-face appointments during prenatal visits or phone calls, to collect information related to maternal health and neonatal outcomes.

Total gestational weight gain (GWG) was calculated by deducting weight at delivery from the pre-conceptional weight and comparing it to the values defined by the Institute of Medicine (IOM). Neonatal characteristics of the participants who delivered at the department of Gynecology and Obstetrics of Hotel-Dieu de France Hospital (HDF), were obtained from the medical charts by the research team on the day of the delivery and classified according to WHO standards (11).

### Dietary assessment

Trained dietitians filled the dietary assessment questionnaires, composed by a validated food frequency questionnaire (FFQ) (Supplementary Table 3) and 24-h dietary recalls. Participants were provided pictures of real food portions to estimate usual quantities of foods consumed. The FFQ was filled during the prenatal visit at the beginning of the third trimester and it included foods characteristic to the Mediterranean eating pattern, to capture the usual intake with more precisions. This FFQ was initially developed and validated in a previous study (12). It was subdivided into 12 categories of food groups: bread and cereals (12 items), rice, pasta, potato and legumes (14 items), milk and dairy products (9 items), fruit and fruit juices (10 items), vegetables (12 items), meat, poultry, fish, eggs and ham (25 items), nuts and condiments (16 items), sugar based sweets, desserts and jams (23 items), bakery products (11 items), salty snacks (3 items), oils and fats (9 items), and beverages (13 items). These subdivisions ended up with a total of 157 food items. The weight in grams of each food was multiplied by its frequency of consumption, and divided, for example, by 7, if it was consumed just once a week. Participants' responses were then converted into average daily intake, in grams.

It is worth to mention that FFQs reflect more the habitual diet over a certain period of time, and cannot predict only the exact composition of nutrients, since it should match with dietary records such as recalls, which are more representative and precise (13, 14). Therefore, in any epidemiological study, a dietary recall should accompany the FFQ to assess the nutritional intake. Concerning the assessment obtained from the three 24-h dietary recalls (each at the beginning of a trimester), participants had to recall all food items and drinks with maximum details possible, consumed the day before, from morning till evening. All interviews during data collection were conducted from Tuesday till Saturday, to avoid recalling the intake of a participant during a weekend, since people usually modify their eating patterns at the end of the week.

Data compiled from the three 24-h dietary recalls and from the FFQ were converted into serving sizes in grams and frequency of consumption and analyzed by a special software (Nutrilog 2.30), to obtain mean daily nutrients and caloric intake estimates. Subjects with extreme values for total energy intake (< 800 Kcal/day or more than 3,500 Kcal/day) were excluded from the study pool.

We collected detailed information on supplement use (date of first use, time, frequency, dose and brand names), however the contribution of micronutrients from dietary supplements to the recall was not included in the analysis, because our focus was mainly on nutrients provided from the daily diet.

### Identification of dietary patterns

DPs are usually assessed either *a priori*—or index-based, when dietary indices are used to determine the adherence to a predefined DP, or *a posteriori*—data-driven, when DPs are statistically identified depending on the reported dietary intake of the participants (15). In this research, this latter technique was applied.

Factorial and cluster analyses are the main techniques applied in nutritional epidemiology to identify the DPs of a population. The research relied on both methods: factorial analysis, to distinguish the inter-correlations between the food groups, and cluster analysis, to assemble the participants into groupings based on similar frequency patterns in food consumption (16, 17).

Twenty three food families with related nutritional characteristics were compiled from the initial pool of the items present in the FFQ as introduced in Supplementary Table 1.

### Statistical analyses

Statistical tests were performed using the SPSS statistical software package version 23. Sample size requirements were calculated by applying the formula published by Tabachnick and Fidell (18). The following took into account: the number of independent variables related to predictable maternal factors included in the model: *N* = 50 + 8 *m* (*m* representing the number of independent variables counted in the study). Those variables were maternal age, educational level, crowding index, profession, pregestational BMI, GWG, smoking habits, and multivitamin supplement intake; given that *m* = 8, at least 114 subjects had to be included in the present study. This formula will assure a statistical power of 80%. Neonatal variables should not be included in the model, since they were unpredictable and unavailable at the time of the recruitment.

After performing a descriptive analysis and showing mean and standard deviation for continuous variables, as well as frequency and percentage of multinomial variables, the population of women was divided according to DPs. The latter were generated according to 2 steps: in step 1, a factor analysis of food items was conducted based on the principal component analysis technique with Kaiser normalization after appropriate checking of assumptions and sample adequacy, followed by a Varimax rotation, since the factors that were output were not correlated. In step 2, the step 1 factors were included in a cluster analysis, by using a K-Means clustering method. The clusters were named and used as DPs in bivariate and multivariable analyses.

Neonatal outcomes were lacking for some participants who did not deliver in HDF. Hence, multiple imputation method, which is a general approach to deal with missing data, was used for neonates' characteristics, using the methods automatically adapting to the missing values types in the analysis. For bivariate analysis, we used the ANOVA to compare means of continuous variables between the three DPs, after checking for normality and homoscedasticity assumptions; these were followed by a *post-hoc* analysis using Bonferroni correction. To compare percentages, a Chi square test was used when calculated results were higher or equal to five; if not so, the Fisher exact test was used. Finally, for multivariate analysis, a MANCOVA was conducted using a General Linear Model, with mother and babies characteristics as continuous dependent variables, dietary patterns as a major independent variable, and other potential confounders for adjustment. In all cases, results were considered significant at a *p* value < 0.05.

## Results

Maternal characteristics and birth outcomes are summarized in Table 1. Three hundred and fifty-eight women participated in the study, with a mean age of 30.54 ± 5.23. Almost three quarters of women (72.1%) had a pre-gestational BMI in the normal range (mean BMI 22.96 ± 3.76), and only 31.8% achieved an adequate GWG, according to the recommended norms of IOM. Sociodemographic data analysis has revealed that almost 88% of the participants had a crowding index equal or < 1, with 60% of them employed and having achieved a university degree (56.7%). Women having a university degree had pregestational BMI values (*p* = 0.015) in the normal range and a crowding index equal or less than 1 (*p* = 0.019), respectively compared to women who attended only high school, showing a statistically significant positive correlations between pregestational BMI and educational and socio-economic levels (data not shown).

**Table 1:** Descriptive characteristics of the sample (N = 358^†^).

**Characteristic**	**Mean**	**SD**
Age (years)	30.54	5.21
Pre-gestational BMI^§^ (kg/m^2^)	22.96	3.76
	**N**	**%**
**Age (years)**		
18–30	197	55
>30	161	45
**Place of residence**		
Beirut	105	29.3
Mount Lebanon	184	51.4
South	25	7
North	20	5.6
Nabatieh	17	4.7
Baalbek	7	2
**Educational level** ^*†*^		
Primary	5	1.4
Complementary	25	7
Secondary	35	9.8
Technical	25	7
Bachelor	203	56.7
Higher education	63	17.6
**Occupation**		
Housewife	112	31.3
Employee	216	60.3
CEO	10	2.8
Unemployed	3	0.8
Freelancer	13	3.6
Intern	2	0.6
Student	2	0.6
**Crowding index** ^*‡*^		
≤ 1	286	88.0
>1	39	12
**Smoking**		
Yes	23	6.4
No	335	93.6
**Pre-gestational BMI** ^§^ **(kg/m** ^**2**^ **)**		
< 18.5	19	5.3
18.5–24.99	258	72.1
≥25	81	22.6
**Gestational weight gain** ^*†*^^**¶**^		
Insufficient	62	17.3
Adequate	114	31.8
Excessive	66	18.4
**Multivitamin supplement use** ^*†*^		
Yes	168	46.9
No	184	51.4

^†^Missing data.

^‡^Calculated as the ratio of total number of co-residents on the numbers of rooms in a household.

^§^Body mass index.

^¶^Gestational weight gain classified according to the norms of IOM (11).

Table 2 presents the neonatal parameters of babies born to women who delivered full term, in the affiliated hospital of USJ. Baby boys had significantly higher anthropometric measurements (weight, height, head circumference) and Apgar scores than baby girls, reflecting gender differences between neonates. Results are shown in Supplementary Table 2.

**Table 2:** Descriptive characteristics of neonatal outcomes of the participants (N = 248^†^).

**Characteristic**	**Mean**	**SD**
**Gestational age (days)**	269.55	10.37
	**N**	**%**
**Mode of delivery** ^†^		
Vaginal	129	36
C-section	116	32.4
**Sex of the child** ^†^		
Female	113	31.6
Male	134	37.4
**Height (cm)** ^†^		
< 46 cm	7	2
46–54 cm	231	64.5
>54 cm	1	0.3
**Weight (kg)** ^†^		
< 2.5 kg	16	4.5
2.5–4 kg	228	63.7
>4 kg	4	1.1
**Head circumference (cm)** ^†^		
< 35 cm	124	34.6
35 cm	58	16.2
>35 cm	54	15.1
**Apgar score (1 min)** ^†^		
< 7	14	3.9
≥7	229	64
**Apgar score (5 mins)** ^†^		
< 7	4	1.1
≥7	239	66.8

^†^Missing data.

### Food categories and consumption patterns

Principal component analysis of the 23 food groups identified the factors of foods consumed with identical frequencies on individual level. Kaiser-Meyer-Olken (KMO) value was 0.754 (*p* value < 0.001 for Bartlett's test of Sphericity), confirming the adequacy of the sample for the analysis. Absolute values < 0.4 were excluded from the table. All food groups are considered to have an interpretable association with a factor. Seven factors, with positive loadings, were derived explaining together 53.056% of the total variance:

- Factor 1 assembled high loadings of Westernized fast food, snacks (chocolate, candies, etc.) and desserts.
- Factor 2 was characterized mainly by fruits, vegetables and seeds.
- Factor 3 had high loadings of rice, pasta, beverages such as coffee and sweetened carbonated choices.
- Factor 4 presented high loadings of lean meats, poultry, fish and plant-based protein sources.
- Factor 5 showed loadings from starchy vegetables and healthy fatty acids (monounsaturated and polyunsaturated lipids).
- Factor 6 was composed of refined cereals (white bread), together with fried and caloric dense Lebanese street foods such as falafel and shawarma, rich in trans and saturated fatty acids, respectively.
- Factor 7 was reserved to eggs and dairy food.

All details are summarized in Table 3.

**Table 3:** Factor loadings of the food groups (FG) (N = 358).

**Food groups**	**FG1**	**FG2**	**FG3**	**FG4**	**FG5**	**FG6**	**FG7**
Westernized fast food	0.740						
Sweets/confectionary	0.702						
Lebanese pies	0.565						
Pastries	0.494						
Salty snacks	0.481						
Fruits/fruit juices		0.681					
Nuts/seeds		0.655					
Vegetables (Raw/cooked)		0.531					
Hot beverages			0.709				
Sweetened beverages			0.660				
Rice/pasta			0.610				
Lean meat				0.752			
Fish				0.636			
Legumes				0.442			
Starchy vegetables					0.715		
MUFA^a^					0.501		
PUFA^b^					0.414		
Refined cereals						0.679	
SFA^c^						0.628	
Lebanese fast food						0.440	
Eggs							0.854
Dairy products							0.550

Extraction Method: Principal Component Analysis; Rotation method: Varimax with Kaiser Normalization; Absolute values < 0.4 were excluded from the table. KMO Value for sample adequacy = 0.754 (P value for Bartlett's test of Sphericity < 0.001). Total variance explained = 53.056%. All food groups are considered to have an interpretable association with a factor.

^*a*^MUFA, monounsaturated fatty acids.

^*b*^PUFA, polyunsaturated fatty acids.

^*c*^SFA, saturated fatty acids.

### Dietary patterns' assessment

A cluster analysis derived from the seven factors led to three main clusters, as presented in Table 4. The nomination of each DP was defined according to the food load of the categories presented in Table 3.

- The first DP was characterized by high consumption of Western-type fast food and Lebanese street foods, with low consumption of fruits, vegetables, seeds, rice, and pasta and was tagged as “Westernized” DP (Cluster 1 with 102 participants).
- The second DP was characterized by moderate consumption of fruits, vegetables, beverages, rice/pasta, and Lebanese street foods, together with a low consumption of proteins, starchy vegetables and healthy fats and was categorized as “ Mixed” DP (Cluster 2 with 99 participants).
- The third DP composed mainly of proteins from lean meat sources, such as poultry and fish, eggs, dairy, fruits, vegetables, legumes and seeds associated with a low consumption of Lebanese street foods, was entitled “Neo-Mediterranean” DP (Cluster 3 with 145 participants). This nomenclature was chosen since the traditional, culture-driven Mediterranean diet pyramid stresses on high intakes of fruits, vegetables, unrefined cereals, legumes and seeds and fish, and moderate consumption of poultry, eggs and dairy (19). However, the younger generation of people living in the Mediterranean region nowadays incorporate more often lean protein sources such as poultry, eggs and dairy, that were consumed in less frequencies by elder generation. This actual food trend led us to propose the connotation of “Neo-Mediterranean.”

**Table 4:** Dietary patterns: cluster analysis of food groups (N = 358).

	**DP1: Westernized**	**DP2: Mixed**	**DP3: Neo-Mediterranean**
	**(N = 102)**	**(N = 99)**	**(N = 145)**
FG1: Westernized fast food and snacks	0.513	*−0.466*	−0.043
FG2: Fruits, vegetables and seeds	*−0.409*	0.394	0.019
FG3: Beverages and rice/Pasta	*−0.559*	0.399	0.121
FG4: Proteins mainly food	*−0.431*	*−0.660*	0.754
FG5: Starchy vegetables and healthy fatty acids	0.548	*−0.615*	0.034
FG6: Westernized Lebanese food	0.333	0.328	*−0.458*
FG7: Eggs and dairy food	−0.013	*−0.355*	0.251

Method: Quick cluster analysis. Number of iterations = 21.

### Correlations of dietary patterns with maternal characteristics

Details concerning the correlations between participants' characteristics and their dietary patterns are presented in Table 5. Significant results were obtained for females adhering to the “Neo-Mediterranean” DP (DP3), who were employed and lived in an urban area (Mount Lebanon), with *p* values ranging from 0.000 to 0.024 respectively. On the other hand, pregnant women following the “Mixed” DP (DP2) had a higher pre-gestational BMI (23.54 ± 4.17) as compared to those adopting the two other DPs (*p* = 0.048).

**Table 5:** Sociodemographic, lifestyle and environmental maternal factors and dietary patterns during pregnancy (N = 358^†^).

**Variables**	**DP1: Westernized**	**DP2: Mixed**	**DP3: Neo-Mediterranean**	***p*-value**
**Sociodemographic**				
**Age (years)**	29.4 ± 5.33	30.94 ± 5.41	30.81 ± 4.82	0.056*
**Place of residence** ^**a, c**^				
Beirut	29 (28.7)	33 (32.7)	39 (38.6)	0.024**
Mount Lebanon	40 (22.6)	54 (30.5)	83 (46.9)	
South	13 (52)	5 (20)	7 (28)	
North	8 (40)	3 (15)	9 (45)	
Nabatieh	10 (58.8)	3 (17.6)	4 (23.5)	
Baalbek	2 (33.3)	1 (16.7)	3 (50)	
**Educational level** ^*†*^				
Primary	3 (60)	1 (20)	1 (20)	0.054**
Complementary	10 (41.7)	7 (29.2)	7 (29.2)	
Secondary	16 (48.5)	8 (24.2)	9 (27.3)	
Technical	9 (36)	4 (16)	12 (48)	
University	54 (27.7)	56 (28.7)	85 (43.6)	
Higher education	10 (16.1)	22 (35.5)	30 (48.4)	
**Occupation** ^**a, b, c**^				
Housewife	50 (46.7)	18 (16.8)	39 (36.4)	0.000**
Employee	45 (21.5)	77 (36.8)	87 (41.6)	
CEO	4 (40)	1 (10)	5 (50)	
Unemployed	1 (33.3)	0 (0)	2 (66.7)	
Freelancer	2 (15.4)	1 (7.7)	10 (76.9)	
Intern	0 (0)	1 (50)	1 (50)	
Student	0 (0)	1 (50)	1 (50)	
**Crowding index**				
≤ 1	78 (27.9)	79 (28.2)	123 (43.9)	0.308**
>1	24 (35.9)	20 (29.7)	22 (34.4)	
**Lifestyle/health behavior**				
**Smoking**				
Yes	9 (40.9)	4 (18.2)	9 (40.9)	0.379**
No	93 (28.7)	95 (29.3)	136 (42)	
**Pre-gestational BMI (kg/m** ^**2**^ **)**	23.15 ± 3.88	23.54 ± 4.17	22.38 ± 3.29	0.048*
**Pre-gestational BMI** ^**b**^				
< 18.5	5 (27.8)	4 (22.2)	9 (50)	0.357**
18.5–24.99	71 (28.4)	68 (27.2)	111 (44.4)	
≥25	26 (33.3)	27 (34.6)	25 (32.1)	
**Gestational weight gain**^**†**^, ¶				
Insufficient	9 (15.8)	21 (36.8)	27 (47.4)	0.977**
Adequate	21 (18.6)	40 (35.4)	52 (46)	
Excessive	11 (17.2)	21 (32.8)	32 (50)	
**Multivitamin supplement intake** ^*†*^				
Yes	42 (25.8)	46 (28.2)	75 (46)	0.246**
No	59 (33.1)	51 (28.7)	68 (38.2)	

^†^Missing data.

*Analysis of variance (ANOVA).

**χ^2^ test.

^¶^Gestational Weight Gain classified according to the norms of IOM (2009).

Categorical variables are presented as N (%) and continuous variables as Mean ± SD.

Correlations are considered significant at p < 0.05.

^a^Significant difference between DP 1 and 2.

^b^Significant difference between DP 2 and 3.

^c^Significant difference between DP 1 and 3.

### Correlations of dietary patterns with neonatal outcomes

Correlations between neonatal outcomes and the three maternal DPs are summarized in Table 6. The “Mixed” DP was associated with a higher gestational age (271.92 ± 6.85days) and with adequate neonatal anthropometric measurements. However, all three maternal DPs did not mediate any significant effect on neonatal outcomes.

**Table 6:** Pregnancy related factors and dietary patterns during pregnancy (N = 358^†^).

**Variables**	**DP1: Westernized**	**DP2: Mixed**	**DP3: Neo-Mediterranean**	***p*-value**
**Gestational age (days)**	268.20 ± 10.54	271.92 ± 6.85	267.64 ± 12.74	0.064*
**Sex of the child**				
Girl	19 (17.3)	47 (42.7)	44 (40)	0.051**
Boy	25 (19.4)	36 (27.9)	68 (52.7)	
**Mode of delivery**				
Vaginal	17 (13.4%)	48 (37.8%)	62 (48.8%)	0.069**
Cesarean	27 (24.5%)	32 (29.1%)	51 (46.4%)	
**Height (cm)**				
< 46 cm	1 (14.3)	1 (14.3)	5 (71.4)	0.462**
46–54 cm	40 (17.9)	80 (35.9%)	103 (46.2)	
>54 cm	0	1 (100)	0	
**Weight (kg)**				
< 2.5 kg	4 (25.0)	4 (25.0)	8 (50.0)	0.804**
2.5–4 kg	39 (17.7)	78 (35.5)	103 (46.8)	
>4 kg	1 (25.0)	1 (25.0)	2 (50.0)	
**Head circumference (cm)**				
< 35 cm	24 (20.2)	40 (33.6)	55 (46.2)	0.445**
35 cm	11 (19.3)	19 (33.3)	27 (47.4)	
>35 cm	5 (9.6)	23 (44.2)	24 (46.2)	
**Apgar score (1 min)**				
< 7	3 (21.4)	6 (42.9)	5 (35.7)	0.710**
≥7	40 (18.1)	76 (34.4)	105 (47.5)	
**Apgar score (5 mins)**				
< 7	0	2 (50.0)	2 (50.0)	0.829**
≥7	43 (18.6)	80 (34.6)	108 (46.8)	

^†^Missing data.

*Resulted from analysis of variance (ANOVA).

**Resulted from X^2^ test.

Categorical variables are presented as N (%) and continuous variables as Mean ± SD; Correlations are considered significant at p < 0.05.

### Correlations of dietary patterns with nutrient intakes

As summarized in Table 7, women following the “Westernized” DP were consuming significantly more calories, fats, sodium, choline and selenium with *p* values below 0.05. On the other hand, nutrient profiles of women adherent to the “Neo-Mediterranean” DP were significantly higher in proteins (*p* = 0.043), fiber (*p* = 0.109), cholesterol (*p* = 0.015) and vitamin A (*p* = 0.014), compared to those following the “Westernized” and the “Mixed” DPs.

**Table 7:** Energy and nutrients intake of the participants across the three dietary patterns (N = 358).

	**DP1: Westernized**	**DP2: Mixed**	**DP3: Neo-Mediterranean**	p*
Energy (Kcal/d)^a^	2,168.95 ± 527.23	1,966.25 ± 554.77	2,045.55 ± 505.93	0.023
CHO (g/day)	272.20 ± 75.97	253.85 ± 80.03	248.78 ± 76.77	0.059
CHO (% EI^+^/d)^b^	50.27 ± 9.86	51 ± 6.18	48.08 ± 5.83	0.005
Protein (g/d)^b^	76.76 ± 21.87	73.86 ± 23.48	81.15 ± 22.71	0.043
Protein (% EI/d)^a, b, c^	13.99 ± 2.12	14.93 ± 2.57	15.93 ± 2.56	0.000
Fat (g/day)^a, c^	92.26 ± 42.58	74.88 ± 22.8	81.06 ± 19.77	0.000
Fat (% EI/d)^a, b^	36.53 ± 5.33	34.22 ± 5.4	35.99 ± 5.26	0.006
Sugar (g/d)	69.33 ± 24.06	166.09 ± 881.12	74.15 ± 27.6	0.247
Fiber (g/d)	17.24 ± 5.84	18.35 ± 8.75	19.29 ± 7.61	0.109
Cholesterol(mg/d)^b^	158.52 ± 74.68	136.32 ± 72.54	163.54 ± 73.63	0.015
Iron (mg/day)	13.72 ± 4.4	14.2 ± 17.4	13.22 ± 4.08	0.748
Calcium (mg/d)	732 ± 322.77	694.98 ± 279.77	745.91 ± 298.19	0.423
Sodium (mg/d)^a, c^	4,863.51 ± 825.35	4,565.76 ± 860.31	4,569.74 ± 798.97	0.011
Vitamin D (μg/d)	8.48 ± 1.87	7.95 ± 1.59	11.38 ± 34.97	0.438
Folate (μg/d)	249.16 ± 98.83	264.65 ± 113.44	313.64 ± 460.06	0.227
Vitamin A (μg/d)^b^	144.77 ± 81.76	117.59 ± 95.23	150.35 ± 87.47	0.014
Vitamin E (μg/d)	9.28 ± 3.52	17.02 ± 85.74	14.53 ± 59.86	0.643
Vitamin C (mg/d)	115.06 ± 65.29	113.98 ± 58.82	104.88 ± 54	0.322
Magnesium (μg/d)^c^	229.64 ± 76.69	258.02 ± 101.6	265.73 ± 104.4	0.013
Zinc (mg/d)	7.09 ± 2.46	7.48 ± 3.15	7.45 ± 2.65	0.512
Iodine (mg/d)	6.96 ± 4.9	8.66 ± 8.76	7.58 ± 6.08	0.190
Choline (mg/d)^a^	208.4 ± 76.06	169.45 ± 81.44	197.79 ± 111.7	0.011
Selenium (μg/d)^a, c^	56.45 ± 20.94	46.98 ± 19.58	47.63 ± 19.38	0.001
Copper (mg/d)	1.3 ± 0.45	1.37 ± 0.48	1.32 ± 0.48	0.543

^*^Analysis of variance One-way ANOVA and Bonferroni *post-hoc* testing, with a p value < 0.05 considered as significant.

^+^EI, energy intake; CHO, carbohydrates; Values are presented as mean ± SD.

^a^Significant difference between DP 1 and 2.

^b^Significant difference between DP 2 and 3.

^c^Significant difference between DP 1 and 3.

### Multivariate analysis of maternal outcomes

The results of multivariate analysis taking separately maternal characteristics (GWG and pre-gestational BMI) as dependent variables, with and without energy adjustments, are presented in Table 8. No significant correlations were highlighted between GWG and maternal socio-demographic variables. However, after energy adjustment, women living in the capital Beirut had a significantly greater GWG than those living in rural regions (β = 0.937; *p* < 0.05). Besides, pre-gestational BMI was positively associated with maternal age (β = 0.156; *p* < 0.05), and inversely with the educational level (β = −0.624; *p* < 0.05). These associations were conserved even after energy adjustment.

**Table 8:** Multivariate analysis of gestational weight gain and pre-gestational BMI of participants.

	**With energy adjustment**			**Without energy adjustment**		
	**Beta**	***p*-value**	**(95% CI)**	**Beta**	***p*-value**	**(95% CI)**
Dependent variable: gestational weight gain						
Age	−0.006	0.525	(−0.024, 0.012)	−0.007	0.446	(−0.026, 0.012)
Education	−0.035	0.510	(−0.139, 0.069)	−0.014	0.794	(−0.121, 0.093)
Crowding index	0.087	0.464	(−0.147, 0.321)	0.134	0.274	(−0.106, 0.373)
DP^†^						
1 vs. 3 (Ref)	0.003	0.980	(−0.259, 0.266)	0.003	0.981	(−0.267, 0.274)
2 vs. 3 (Ref)	−0.034	0.753	(−0.247, 0.179)	−0.056	0.614	(−0.276, 0.163)
Residence^§^						
1 vs. 6 (Ref)	0.937	0.030	(0.093, 1.780)	0.832	0.060	(−0.036, 1.700)
2 vs. 6 (Ref)	0.807	0.056	(−0.022, 1.637)	0.695	0.110	(−0.158, 1.548)
3 vs. 6 (Ref)	0.915	0.060	(−0.038, 1.868)	0.775	0.120	(−0.204, 1.754)
4 vs. 6 (Ref)	0.706	0.118	(−0.180, 1.592)	0.631	0.175	(−0.282, 1.543)
5 vs. 6 (Ref)	0.641	0.233	(−0.416, 1.697)	0.441	0.424	(−0.643, 0.524)
Energy	0.000	< 0.001	(0.000, 0.001)	–	–	–
Dependent variable: pre–gestational BMI						
Age	0.153	0.002	(0.059, 0.248)	0.156	0.001	(0.061, 0.250)
Education	−0.588	0.032	(−1.125, −0.050)	−0.624	0.023	(−1.160, −0.088)
Crowding index	−0.244	0.691	(−1.452, 0.965)	−0.326	0.594	(−1.531, 0.878)
DP						
1 vs. 3 (Ref)	0.223	0.746	(−1.132, 1.578)	0.223	0.746	(−1.134, 1.581)
2 vs. 3 (Ref)	1.056	0.060	(−0.046, 2.158)	1.095	0.051	(−0.007, 2.197)
Residence						
1 vs. 6 (Ref)	3.489	0.117	(−0.879, 7.840)	3.666	0.099	(−0.692, 8.023)
2 vs. 6 (Ref)	3.053	0.162	(−1.236, 7.342)	3.253	0.136	(−1.033, 7.538)
3 vs. 6 (Ref)	4.520	0.072	(−0.404, 9.443)	4.769	0.057	(−0.148, 9.686)
4 vs. 6 (Ref)	3.687	0.114	(−0.893, 8.267)	3.821	0.102	(−0.762, 8.405)
5 vs. 6 (Ref)	2.475	0.373	(−2.985, 7.934)	2.830	0.307	(−2.612, 8.272)
Energy	−0.001	0.192	(−0.002, 0.000)	–	–	–

^†^Dietary Pattern 1: Westernized, 2: Mixed, 3: Neo-Mediterranean.

^§^Residence 1: Beirut, 2: Mount-Lebanon, 3: South, 4: North, 5: Nabatiyeh, 6: Baalbek.

*Significant p value (< 0.05) in bold.

### Multivariate analysis of neonatal outcomes

The results of multivariate analysis considering each neonatal parameter as a dependent variable, with and without energy adjustments and missing values replacement, are presented in Tables 9–12. First, maternal factors were not correlated with the neonates' length. But, following the missing values replacement, this parameter was negatively associated with maternal age (β = −0.004; *p* < 0.05) and positively with pre-gestational BMI (β = 0.007; *p* < 0.05). This association was conserved independently of the energy adjustment.

**Table 9:** Multivariate analysis of neonatal outcomes.

**Variables**		**Height**	**Weight**	**Head circumference**	**Apgar test (1 min)**	**Apgar test (5 mins)**
**No missing value replacement; with energy adjustment** ^¶^						
Smoking	Yes vs. No (Ref.)	0.02	0.015	**−0.513**	0.057	**−0.075**
		(−0.092 to 0.132)	(−0.148 to 0.178)	**(−0.993 to** −0.033)*	(−0.086 to 0.199)	**(−0.145 to** −0.005)*
DP^†^	1 vs. 3 (Ref.)	0.003	−0.37	−0.124	−0.007	0.023
		(−0.067 to 0.073)	(−0.139 to 0.064)	(−0.423 to 0.175)	(−0.095 to 0.082)	(−0.02 to 0.067)
	2 vs. 3 (Ref.)	0.029	−0.38	0.056	−0.005	−0.009
		(−0.028 to 0.086)	(−0.121 to 0.045)	(−0.189 to 0.301)	(−0.078 to 0.068)	(−0.045 to 0.026)
Age		−0.004	−0.006	−0.006	0.000	**−0.005**
		(−0.009 to 0.001)	(−0.13 to 0.001)	(−0.027 to 0.016)	(−0.007 to 0.006)	**(−0.008 to** −0.002)*
Education level		0.006	−0.004	**0.213**	0.002	−0.005
		(−0.022 to 0.033)	(−0.45 to 0.036)	(0.094 to 0.332)*	(−0.034 to 0.037)	(−0.023 to 0.012)
Crowding index		−0.011	0.009	**0.275**	−0.025	−0.01
		(−0.071 to 0.049)	(−0.079 to 0.097)	(0.017 to 0.533)*	(−0.102 to 0.051)	(−0.048 to 0.027)
Pre-gestational BMI		0.006	**0.011**	0.004	−0.002	0.003
		(−0.002 to 0.013)	(0.000 to 0.021)*	(−0.027 to 0.035)	(−0.011 to 0.007)	(−0.001 to 0.008)
Gestational weight gain		0.011	**0.060**	**0.25**	−0.023	**−0.033**
		(−0.026 to 0.048)	(0.006 to 0.114)*	(0.091 to 0.409)*	(−0.071 to 0.024)	**(−0.056 to** −0.01)*
Energy intake (Kcal)		−1.431E−5	**−8.108E−5**	2.011E−5	−6.263E−5	−1.740E−5
		(−6.468E−5 to 3.607E−5)	**(0.000 to** −7.548E−6)*	(0.000 to 0.000)	(0.000 to 1.676E−6)	(−4.883E−5 to 1.404E−5)
Gestational age (days)		5.883E−5	0.000	0.000	0.000	0.000
		(0.000 to 0.000)	(−9.668E−5 to 0.001)	(−0.001 to 0.001)	(0.000 to 0.000)	(−4.382E−5 to 0.000)

^¶^Sex of the child and delivery mode were removed from the analysis because there were not correlated with neonatal outcomes.

^†^Dietary Pattern 1: Westernized; 2: Mixed; 3: Neo-Mediterranean.

*Significant p value (< 0.05) in bold.

**Table 10:** Multivariate analysis of neonatal outcomes.

**Variables**		**Height**	**Weight**	**Head circumference**	**Apgar test (1 min)**	**Apgar test (5 mins)**
**No missing value replacement; without energy adjustment** ^¶^						
Smoking	Yes vs. no (Ref.)	0.022	0.028	**−0.516**	0.067	**−0.072**
		(−0.089 to 0.134)	(−0.137 to 0.192)	**(−0.994 to** −0.038)*	(−0.077 to 0.21)	**(−0.142 to** −0.003)*
DP^†^	1 vs. 3 (Ref.)	0.003	−0.037	−0.124	−0.006	0.024
		(−0.066 to 0.073)	(−0.14 to 0.065)	(−0.423 to 0.174)	(−0.096 to 0.083)	(−0.02 to 0.067)
	2 vs. 3 (Ref.)	0.03	−0.035	0.055	−0.003	−0.009
		(−0.027 to 0.087)	(−0.119 to 0.049)	(−0.189 to 0.299)	(−0.076 to 0.071)	(−0.044 to 0.27)
Age		−0.004	−0.006	−0.006	−0.001	**−0.005**
		(−0.009 to 0.001)	(−0.014 to 0.001)	(−0.027 to 0.016)	(−0.007 to 0.006)	**(−0.008 to** −0.002)*
Education level		0.006	−0.005	**0.213**	0.002	−0.005
		(−0.022 to 0.033)	(−0.045 to 0.036)	(0.095 to 0.332)*	(−0.034 to 0.037)	(−0.023 to 0.012)
Crowding index		−0.011	0.012	**0.274**	−0.023	−0.01
		(−0.071 to 0.049)	(−0.077 to 0.1)	(0.017 to 0.532)*	(−0.1 to 0.054)	(−0.047 to 0.028)
Pre-gestational BMI		0.006	**0.013**	0.003	0.000	0.004
		(−0.001 to 0.013)	(0.003 to 0.024)*	(−0.027 to 0.033)	(−0.009 to 0.009)	(0.000 to 0.008)
Gestational weight gain		0.009	0.044	**0.254**	−0.035	**−0.036**
		(−0.027 to 0.044)	(−0.009 to 0.097)	(0.1 to 0.407)*	(−0.081 to 0.011)	**(−0.059 to** −0.014)*
Gestational age (days)		4.192E−5	0.000	0.000	0.000	8.154E−5
		(0.000 to 0.000)	(0.000 to 0.000)	(−0.001 to 0.001)	(−0.001 to 7.301E−5)	(−5.963E−5 to 0.000)

^¶^Sex of the child and delivery mode were removed from the analysis because there were not correlated with neonatal outcomes.

^†^Dietary Pattern 1: Westernized; 2: Mixed; 3: Neo-Mediterranean.

*Significant p value (< 0.05) in bold.

**Table 11:** Multivariate analysis of neonatal outcomes.

**Variables**		**Height**	**Weight**	**Head circumference**	**Apgar test (1 min)**	**Apgar test (5 mins)**
**Missing value replacement; with energy adjustment** ^¶^						
Smoking	Yes vs. No (Ref.)	0.001	0.047	**−0.478**	**0.068**	**−0.055**
		(−0.051 to 0.054)	(−0.019 to 0.113)	**(−0.662 to** −0.293)*	(0.013 to 0.123)*	**(−0.088 to** −0.022)*
DP^†^	1 vs. 3 (Ref.)	0.002	−0.03	−0.085	−0.001	**0.031**
		(−0.031 to 0.034)	(−0.071 to 0.011)	(−0.2 to 0.029)	(−0.036 to 0.033)	(0.01 to 0.051)*
	2 vs. 3 (Ref.)	0.02	−0.02	0.022	−0.013	−0.013
		(−0.008 to 0.047)	(−0.055 to 0.014)	(−0.074 to 0.118)	(−0.042 to 0.016)	(−0.03 to 0.004)
Age		**−0.004**	**−0.006**	−0.007	−0.001	**−0.005**
		**(−0.006–** −0.002)*	**(−0.009 –** −0.003)*	(−0.015 to 0.002)	(−0.004 to 0.001)	**(−0.006 to** −0.003)*
Education level		0.002	0.01	**0.196**	0.000	−0.002
		(−0.012 to 0.015)	(−0.006 to 0.027)	(0.149 to 0.242)*	(−0.014 to 0.014)	(−0.011 to 0.006)
Crowding index		−0.003	−0.006	**0.29**	**−0.041**	**−0.026**
		(−0.032 to 0.026)	(−0.042 to 0.031)	(0.188 to 0.391)*	**(−0.072 to** −0.011)*	**(−0.044 to** −0.008)*
Pre-gestational BMI		**0.007**	**0.008**	0.006	−0.001	**0.003**
		(0.004 to 0.011)*	(0.003 to 0.012)*	(−0.006 to 0.018)	(−0.005 to 0.002)	(0.001 to 0.005)*
Gestational weight gain		0.012	**0.058**	**0.245**	**−0.033**	**−0.04**
		(−0.006 to 0.03)	(0.035 to 0.08)*	(0.183 to 0.308)*	**(−0.051 to** −0.014)*	**(−0.052 to** −0.029)*
Energy (Kcal)		−1.246E−5	**−8.210E−5**	3.933E−5	**−4.956E−5**	−7.726E−6
		(−3.621E−5–1.128E−5)	**(0.00–** −5.225E−5)*	(−4.40E−5 to 0.000)	**(−7.453E−5 to** −2.458E−5)*	(−2.259E−5 to 7.136E−6)
Gestational age (days)		3.663E−5	**0.000**	0.000	**0.000**	**0.000**
		(−7.48E−5 to 0.00)	(0.000 to 0.000)*	(0.000 to 0.001)	**(0.000 to** −7.69E−6)*	(5.177E−5 to 0.000)*

^¶^ Sex of the child and delivery mode were removed from the analysis because they were not correlated with neonatal outcomes.

^†^Dietary Pattern 1: Westernized; 2: Mixed; 3: Neo-Mediterranean.

*Significant p value (< 0.05) in bold.

**Table 12:** Multivariate analysis of neonatal outcomes.

**Variables**		**Height**	**Weight**	**Head circumference**	**Apgar test 1 min**	**Apgar test 5 mins**
**Missing value replacement; without energy adjustment** ^¶^						
Smoking	Yes vs. no (Ref.)	0.003	0.057	**−0.482**	**0.074**	**−0.054**
		(−0.049 to 0.055)	(−0.01 to 0.124)	**(−0.666 –** −0.299)*	(0.019 to 0.129)*	**(−0.087 to** −0.021)*
DP^†^	1 vs. 3 (Ref.)	0.001	−0.032	−0.084	−0.003	**0.03**
		(−0.031 to 0.034)	(−0.074 to 0.009)	(−0.199 to 0.03)	(−0.037 to 0.032)	(0.01 to 0.051)*
	2 vs. 3 (Ref.)	0.02	−0.016	0.02;	−0.01;	−0.012;
		(−0.007 to 0.048)	(−0.051 to 0.019)	(−0.076 to 0.116)	(−0.039 to 0.018)	(−0.029 to 0.005)
Age		**−0.004;**	**−0.006; –**	−0.007;	−0.001;	**−0.005;**
		**(−0.006 to** −0.002)*	**(0.009 to** −0.003)*	(−0.015 to 0.002)	(−0.003 to 0.001)	**(−0.006 to** −0.003)*
Education level		0.002;	0.01;	**0.196;**	−6.416E−6;	−0.002;
		(−0.012 −0.015)	(−0.007 to 0.027)	(0.15 to 0.243)*	(−0.014 to 0.014)	(−0.011 to 0.006)
Crowding index		−0.003;	−0.002	**0.288**	**−0.039**	**−0.026**
		(−0.032 to 0.026)	(−0.039 to 0.034)	(0.187 to 0.389)*	**(−0.07 to** −0.009)*	**(−0.044 to** −0.008)*
Pre-gestational BMI		**0.008**	**0.01**	0.005	−4.228E−5	**0.004**
		(0.004 to 0.011)*	(0.006 to 0.14)*	(−0.007 to 0.017)	(−0.004 to 0.004)	(0.001 to 0.006)*
Gestational weight gain		0.01	**0.043**	**0.252**	**−0.042**	**−0.042**
		(−0.007 to 0.027)	(0.021 to 0.065)*	(0.191 to 0.313)*	**(−0.06 to** −0.023)*	**(−0.053 to** −0.031)*
Gestational age (days)		2.200E−5	**0.000**	0.000	**0.000**	**0.000**
		(−8.59E−5 −0.00)	(2.09E−5 to 0.00)*	(0.000 to 0.001)	**(0.000 to** −6.90E−5)*	(4.492E−5 to 0.000)*

^¶^ Sex of the child and delivery mode were removed from the analysis because they were not correlated with neonatal outcomes.

^†^Dietary Pattern 1: Westernized; 2: Mixed; 3: Neo-Mediterranean.

*Significant p value (< 0.05) in bold.

With each one-unit increase in pre-gestational BMI, neonate's weight increased by 1.3% (β = 0.013; *p* < 0.05). In addition, when the analysis was adjusted for energy intake, the weight was directly associated with the maternal pre-gestational BMI (β = 0.011) and the GWG (β = 0.060), and inversely associated with the energy intake (β = −8.108E−5). After missing values replacement, this neonatal outcome was positively associated with the pre-gestational BMI (β = 0.008) and the GWG (β = 0.058) but negatively correlated with the maternal age (β = −0.006) and energy intake (β = −8.210E−5).

Neonates' head circumference was inversely associated with maternal smoking (β = −0.516), and directly associated with maternal educational level (β = 0.213), their crowding index (β = 0.274) and their GWG (β = 0.254); all these correlations were significant (*p* < 0.05) and were conserved even after energy adjustment. Following the missing values replacement, this parameter remained positively associated with the professional status, the educational level, the crowding index and the GWG, but was lower in neonates whose mothers were smoking before or during the pregnancy (β = −0.478). These results were maintained after energy adjustment.

No significant correlations were observed between the Apgar score at 1 minute and maternal socio-demographic variables, with or without energy adjustment. However, after missing values replacement and energy adjustment, this variable was higher in smoking mothers but was inversely associated with the crowding index (β = −0.041), the GWG (β = −0.033) and the total energy intake (β = −4.956E−5). These correlations were sustained even without energy adjustment.

Finally, an inverse association was significantly noted between the Apgar score at 5 mins and maternal smoking (β = −0.072; *p* < 0.05) as well as with maternal age (β = −0.005; *p* < 0.05) and GWG (β = −0.036; *p* < 0.05). These associations were preserved after energy adjustment. In addition to these correlations and after missing values replacement, the Apgar score at 5 mins was higher in mothers adopting the “Westernized” DP (β = 0.031), compared to those adopting the “Neo-Mediterranean” one, and it was positively associated with the pre-gestational BMI (β = 0.003), and negatively with the crowding index (β = −0.026). These correlations were conserved even without energy adjustment.

## Discussion

The present research is the first study evaluating the DPs of pregnant females, in a Middle-Eastern country of the Mediterranean basin, correlated to maternal and neonatal parameters. In our sample, three DPs were detected: the Westernized, Mixed and Neo-Mediterranean patterns. DPs did not have any significant impact on maternal and neonatal outcomes, thus rejecting our hypothesis. The percentage of variance by the three DPs was found to be 53%, higher than the results of 31% reported by Itani et al. (20), in a study among pregnant Emirati women.

Frequent consumption of fast food, associated with low ingestion of fruits, vegetables, seeds and protein sources was identified as the ”Westernized” DP. The dietary intakes of women adherent to this profile depict higher caloric intake, associated with a higher consumption of carbohydrates, fats and sodium, and lower intakes of fiber, folate, vitamin E, magnesium, and iodine. This can be explained by the frequent inclusion of fast food (Western and Lebanese types) in their meal plan, thus potentially contributing to energy dense choices, low in crucial nutrients such as folate and high in lipids and salt. Our results are comparable to those obtained by Arkkola et al. (21), where higher intakes of calories, lipids and sugars were dominant in pregnant women following the “Fast Food” DP.

The “Mixed” DP was based on a frequent consumption of healthy food choices (fruits, vegetables, seeds) combined with Westernized ones (Lebanese street foods), thus the mean dietary intakes of those following this eating pattern showed high loads of carbohydrates, sugars, iron, vitamin E, zinc and iodine with low levels of cholesterol and vitamin D. The inclusion of those food groups enhanced the overall diet quality of women following this dietary profile. Statistical analysis identified that those following the DP “Mixed,” were females having initially a higher pre-gestational BMI. This issue is explained by the fact that during pregnancy, women with higher pre-gestational BMI become more motivated than others to adopt a healthier lifestyle, especially regarding food patterns, to counterbalance the negative influence of excess weight and unbalanced food choices on gestational outcomes (22). Thus, in our sample, those initially having higher BMI values tried to include in their daily diets fruits, vegetables and seeds more often than before, in addition to consuming rice, pasta and some traditional fast-foods. In a recent publication conducted among Emirati pregnant women, those adherent to the “Diverse” DP were older than 30 years old and had a higher income, compared with those following the Western DP (20). Our results confirm that mean maternal age was the highest among those having a “Mixed” DP. As for the socioeconomic status, it was determined by the crowding index and no direct question was addressed to collect data on income, because usually participants are reluctant to share their financial status.

On the other hand, the majority of our participants were adherent to the “Neo-Mediterranean” DP, represented mostly by protein based choices, similar to the “High protein” DP described by Grieger et al. (23). It was characterized by high loads of proteins, fibers, cholesterol, calcium, vitamins A and D, folate and magnesium, due to the fact of frequent intakes of eggs, milk and dairy products, and lean meat choices. Women following this regimen had a higher educational level and initially a lower pre-gestational BMI, as compared to the other subgroups of the sample. Living in urban areas and being employed were some maternal factors significantly correlated to this particular DP. Our results join the ones of Arkkola et al. (21), where educated, older, employed and having a higher income pregnant women had a “Healthy” DP, compared to other groups.

Starting pregnancy with an excess body weight and GWG influences directly maternal and neonatal outcomes (24). In this sample, a BMI higher than 25 kg/m^2^ was positively associated with maternal age and inversely with the educational level. In a large US cohort, pregnant women with initially excess body weight were mostly adherent to “Westernized” DP (25), unlike our results where those following the “Mixed” and “Neo-Mediterranean” DPs had respectively the highest and the lowest pre-gestational BMI. The reason behind is the low incidence of underweight, overweight and obesity in the sample, with only 18% of participants having a BMI higher than 25 kg/m^2^. As for GWG, the results of Rohatgi et al. (26) reported significant links between maternal age and the adoption of “Westernized” diets with weight gain. Unlike our results, where no significant associations were observed, probably because the majority of our participants respected the recommendations, and few gained below or above the norms. In addition, weight gain was directly correlated with energy intake, thus eating more was responsible for putting on more weight. No significant associations were observed between the DPs, maternal weight gain and pre-gestational BMI in our sample. The cause may be the dispersion of both nutritious and energy dense foods among the “Westernized” and the “Mixed” DPs, which decreased the force of this association.

Fetal nicotine exposure impairs oxygen availability and normal placental nutrient transfer; this may decrease birth weight by 150–200 gm, contribute to intrauterine growth retardation, prematurity and small-for-gestational age infants, compared to not exposed neonates (27). In our research, smoking was among the maternal factors leading to adverse neonatal outcomes reflected on head circumference and Apgar score at 5 mins, but not on birth weight.

Concerning neonatal outcomes, in a recent review, Raghavan et al. (28) concluded that “Healthy” DPs (higher in fruits, vegetables, whole grains, legumes, nuts and seeds) during pregnancy were associated with a lower risk of preterm deliveries, but no clear conclusion was made with regards to the impact of any DP, before or during pregnancy, on birth outcomes. In our research, no significant differences were detected pertaining to gestational age and the three DPs, unlike the results of Martin et al. (29) that revealed that the risk of preterm delivery was greater in those having a poor diet quality. Besides, according to Grieger et al. (23), a “High protein” DP reduced the risk premature birth in a sample of Australian women.

Hu et al. identified that adherence to a “Fast Food” DP, rich in fat and sugar by pregnant females, induced adverse postnatal outcomes in offspring, leading to a rapid weight gain during childhood (30). On the other hand, Japanese women in the lowest nutritional adequacy pattern (Wheat products) gave birth to offsprings with significantly lower birth weight and head circumference, unlike those following the “Meat and egg” and “Rice, fish and vegetables” patterns (31). In a Danish prospective study, women in the “Health conscious” profile had lower risk of small birth weight than those in the “Western diet” cluster (32). In contrast, neither the adherence to a “Mediterranean” DP (33), nor the “Mixed” DP had an impact on fetal growth and birth outcomes (34). According to other studies in the field, higher intake of fruits, vegetables and lean protein sources reduces the risk of negative birth outcomes (31, 32, 35). In the present research, no significant associations were mediated between neonatal outcomes and the three DPs, similar to the study of Xie et al. (36). The inconsistency of our results could be explained by the fact that all participants had full term deliveries, with no gestational complications and, thus, infants were generally born at a healthy weight. In addition, no single DP contained only fruits, vegetables and lean proteins in our analyses, because they were dispersed between the “Mixed” and the “Neo-Mediterranean” classes.

Furthermore, after adjusting for potential confounders, the inclusion of maternal factors in the multivariate models did not change their associations with offspring's outcomes. The reason behind may be due to the distribution of pre-gestational BMI and GWG in the acceptable norms.

Our neonatal outcomes were not significantly correlated with any of the studied DPs, similar to the results of Colon-Ramos et al. (34) and of Saunders et al. (33). Unlike our results, in a recent publication, women adopting “Westernized” DPs gave birth to small-for-age infants with smaller head circumference (37). It should be noted that among our participants, those adopting the “Neo-Mediterranean” eating pattern gave birth to infants with better anthropometric outcomes, within WHO norms. This highlights the impact of good quality food, among the multiple factors interfering with genetic and environmental cues, on fetal development.

Even though Lebanese cuisine is reputed for its taste, variety and having common basis with the Mediterranean diet, a major drawback from this study is the fact that we couldn't identify a Mediterranean DP representing separately this healthy model, among our sample. This underlines the ongoing nutrition mutation affecting traditional societies, that shifts toward a modernized cuisine and the adoption of westernized dietary profiles with low quality nutrients. However, our finding of a “Neo-Mediterranean” DP, composed of lean sources and mostly from dairy products may represent the actual tendency of a healthy move of our traditional Mediterranean pattern.

Potential limitations of this research are due to the observed associations that don't represent causal effects, but are related to the nature of this observational study. In addition, misclassification of outcomes is a limitation that needs to be reported, since dietary intake was compiled retrospectively. Despite the fact that a follow-up was kept with the participating women until delivery, some did not agree to share with the research team data related to neonatal outcomes and this explains some missing data. However, this drawback was corrected by using the statistical method of value prediction. Another limitation concerns the subjective labeling by the research team of the DPs, derived from factor analysis, since some patterns with similar content, have an alternative designation in other publications (38). However, the selection of those food groupings were in accordance to other studies published worldwide (20, 28, 39). Finally, although the recruitment was done in different regions of the country and the high follow-up rate until delivery, the participants were not a representative sample of the Lebanese women, thus, the present findings cannot be generalized, and should be conducted on a larger scale, by applying proper sampling strategies. In addition, no clear distinction regarding the classification of the regions according to their urban or rural settings was taken into consideration from the start of the study.

On the other hand, the added value of this paper is the use of a validated FFQ, created by the main authors and filled by well-trained dietitians. This contributed to the strengths of the results, since tools assessing nutrient intake should be culturally adapted. Seasonality of food selection was taken into consideration by interviewing participants once at the beginning of each trimester of gestation. In addition, maternal and neonatal parameters were compiled by the research team, in order not to end-up with irrelevant information, and dietary data collection and nutrient analysis were performed by the same person. To minimize the effect of under or mis-reporting of food intakes, analyses with and without energy adjustment were conducted to limit measurement bias. Finally, to our knowledge, this research is the first assessing the DP of Arab women in the Middle-East and living in the Mediterranean basin.

## Conclusion

Although DPs are culture sensitive, population specific and help capturing the complexities of a diet (30), the three DPs identified in the present study were not exclusively compatible with the traditional Mediterranean dietary pattern, as expected among those living in the Mediterranean region. The categorized DPs in this research were in correlation with pre-gestational BMI and other socio-demographic maternal variables, such as age and educational level, but did not impact infants' birth characteristics. Future research should aim the inclusion of a larger sample, on national level, to achieve a global picture on the impact of maternal nutrition on gestational and neonatal outcomes.

Nevertheless, knowing that adequate nutritional status is vital during this critical period of life, public health policies, under the auspices of the government, should reinforce awareness campaigns among women in child-bearing age, in order to optimize nutrients intakes during pregnancy, while incorporating healthier food choices with more ingredients from the traditional Mediterranean cuisine.

## Data availability statement

The original contributions presented in the study are included in the article/Supplementary material, further inquiries can be directed to the corresponding author.

## Ethics statement

The studies involving human participants were reviewed and approved by Institutional Review Board of Saint-Joseph University at Beirut Lebanon, the Hotel-Dieu Hospital Ethics Committee (CE HDF624/FP49). The patients/participants provided their written informed consent to participate in this study.

## Author contributions

TP conceived the study, supervised the recruitment of the participants, and prepared the manuscript. PS conducted the statistical analysis. GA and AK contributed to the design of the study. CA contributed to the design of the study and in the preparation of the manuscript. MA did the interviews and arranged the data. LR provided complete supervision, critical revision, data interpretation, and correction of the manuscript. All authors constituted the research team.

## Funding

This research received a grant from the Research Council of Saint-Joseph University (FP49/Nov 2014).

## Conflict of interest

The authors declare that the research was conducted in the absence of any commercial or financial relationships that could be construed as a potential conflict of interest.

## Publisher's note

All claims expressed in this article are solely those of the authors and do not necessarily represent those of their affiliated organizations, or those of the publisher, the editors and the reviewers. Any product that may be evaluated in this article, or claim that may be made by its manufacturer, is not guaranteed or endorsed by the publisher.

## Abbreviations

BMI: body mass index
DP: dietary pattern
FFQ: Food Frequency Questionnaire
FG: food group
GWG: gestational weight gain
HDF: Hôtel Dieu de France
IOM: Institute of Medicine
KMO: Kaiser-Meyer-Olken
MUFA: monounsaturated fatty acids
PUFA: polyunsaturated fatty acids
SFA: saturated fatty acids
SD: standard deviation
USJ: Saint-Joseph University
WHO: World Health Organization.
