# Supplementary material for: Dietary patterns and birth outcomes of healthy Lebanese pregnant women
Source: Front Nutr. 2022 Sep 27;9:977288. doi: 10.3389/fnut.2022.977288 (PMC9551999; doi:10.3389/fnut.2022.977288)
Supplement: Supplementary file 1 [file Data_Sheet_1.PDF]

<u>القسم الثاني:</u> استمارة عن غذاء المرأة الحامل (أسئلة حول الإستهلاك اليومي للأطعمة)

	الطعام	الحصة	عدد الحصص	عدد مرّات الإستهلاك				
				في اليوم	في الأسبوع	في الشهر	خلال فترة الحمل	أبدًا
A	**الخبز والحبوب**							
1	الخبز العربي (منها السندويشات)	**¼ رغيف كبير<br>½ رغيف وسط**						
2	الخبز المرقوق / خبز التنّور	**1 تنور<br>½ مرقوق**						
3	خبز الهمبرغر	**1 قطعة**						
4	خبز فرنجي (باغيت)	**30 غ<br>أو حجم صغير**						
5	خبز بالحليب (Pain au lait)	**1 قطعة**						
6	توست، كراكوت	**3 قطع**						
7	بان دومي	**قطعتين**						
8	كعك	**8 قطع مدوّرة<br>3 أصابع وسط<br>1 إصبع كبير**						
	ما هو نوع هذه المأكولات؟	☐ أبيض		☐ أسمر (قمحة كاملة)			☐ غير ذلك:	
9	كورن فلايكس عادي	**½ كوب**						
10	كورن فلايكس غنيّ بالألياف	**¾ كوب**						
11	لوح حبوب (Barre de Céréales)	**30 غ<br>أو 1 قطعة**						
12	شوفان	**½ كوب**						
B	**البطاطا والمعكرونة والأرز والحبوب**							
13	أرز مطبوخ	**كوب<br>(15 معلقة كبيرة)**						
14	محاشي	**12 ورق عنب<br>6 ملفوف 3 كوسى**						
15	برغل مطبوخ / فريكة مطبوخة / مغربية	**كوب<br>(10 ملاعق كبيرة)**						
16	كبّة	**1 قطعة وسط<br>أو قرصين**						
17	معكرونة أو باستا مطبوخة	**كوب**						
18	بطاطا (منها المهروسة أو بوريه)	**١ قطعة وسط<br>أو كوب**						
19	بطاطا مقلية	**10 -12 قطعة**						

20	كستناء	**4 – 5 قطع**						
	**الطعام**	**الحصة**	**عدد الحصص**	**عدد مرّات الإستهلاك**				
				**في اليوم**	**في الأسبوع**	**في الشهر**	**خلال فترة الحمل**	**أبدًا**
21	كينوة	**5 ملاعق كبيرة**						
22	بازيلا	**½ كوب<br>(5 ملاعق كبيرة)**						
23	ذرة	**½ كوب<br>(5 ملاعق كبيرة)**						
24	فول، فاصوليا	**كوب<br>(10 ملاعق كبيرة)**						
25	عدس	**كوب<br>(10 ملاعق كبيرة)**						
26	حمص	**كوب<br>(10 ملاعق كبيرة)**						
**C**	**الحليب ومشتقاته**							
27	حليب (بما في ذلك في القهوة أو مع الكورن فلايكس)	**كوب<br>أو 240 مل**						
	نوع الحليب	☐ كامل الدسم			☐ نصف دسم			☐ خالي من الدسم
28	لبن (بما في ذلك في الأطباق المطبوخة)	**كوب<br>أو 240 مل**						
	نوع اللبن	☐ كامل الدسم			☐ نصف دسم			☐ خالي من الدسم
29	لبنة	**50 غ<br>أو 3 ملاعق كبيرة**						
	نوع اللّبنة	☐ كاملة الدسم			☐ نصف دسم			☐ خالية من الدسم
30	أجبان %10> دهون<br>(ex. Picon light, Présilège, Sylphide …)	**قطعتين**						
31	أجبان مثل:<br>بلغاري بقر، شنكليش، دوبل كريم، حلوم "دايت"، موزاريل ابيضاء، بارميزان، بلدي	**30 غ أو<br>شرحتين رفيعتين**						
32	أجبان مثل:<br>بلغاري غنم، فيتا، حلوم، ريكوتا، موزاريلا صفراء، مجدولة	**30 غ أو<br>شرحتين رفيعتين**						
33	أجبان مثل:<br>بورسين، شيدار، فوندال، جبن الماعز، غروبير، قشقوان، كيري، لا فاش كيري، بيكون، سميدس	**30 غ أو<br>شرحتين رفعتين**						
34	قريشة	**ملعقتين كبيرتين**						
35	كشك	**½ كوب**						

	الطعام	الحصة	عدد الحصص	عدد مرّات الإستهلاك				
				في اليوم	في الأسبوع	في الشهر	خلال فترة الحمل	أبدًا
D	**الفاكهة وعصير الفاكهة**							
36	خرما،موز، تفاح، ليمون، إجاص، دراق، نكتارين	**حبّة صغيرة**						
37	مشمش، خوخ، أفندي، تين، كيوي، صبيّر، بلح	**حبّتين صغيرتين**						
38	كرز، عنب، فريز، أكيدنيا	**12 حبّة**						
39	قشطة ، مانغا	**⅓ حبّة**						
40	بطيخ، شمّام	**شرحة**						
41	سلطة فاكهة، رمان، توت، كومبوت فاكهة	**½ كوب**						
42	فاكهة مجفّفة (مثل: مشمش، زبيب، تمر، تين)	**3-2 حبّة أو معلقة كبيرة**						
43	عصير الفاكهة المعلّب	**كوب**						
44	عصير الفاكهة الطّازج	**كوب**						
45	مشروبات اصطناعية بنكهة الفاكهة (تانغ، دارينا...)	**كوب**						
E	**الخضار**							
46	بندورة، خيار، خس، ملفوف، جزر نيّ	**كوب**						
47	نعنع، روكا، بقلة، فجل، بصل أخضر، زعتر، فليفلة	**كوب**						
48	لوبية ، بامية، أرضي شوكي	**كوب**						
49	سبانخ، سلق، ملوخية	**كوب**						
50	كوسى، شمندر، جزر، قرنبيط،، بروكولي (مسلوق)	**كوب**						
51	قرنبيط، كوسى (مقليّ)	**2 – 3 قطع**						
52	فول اخضر، بازيلا خضراء	**كوب**						
53	باذنجان	**½ كوب**						
	طريقة التحضير	☐ مشويّ			☐ مقليّ			☐ بالطحينة
54	صلصة البندورة	**½ كوب**						

55	فتوش، تبولة	**كوب**						
56	شوربة خضار	**كوب**						
57	مكدوس، كبيس، خضروات مخلّلة	**قطعتين**						
**F**	**اللحوم، الأسماك والبيض**							
58	دجاج	**90 غ**						
	**الطعام**	**الحصة**	**عدد الحصص**	**عدد مرّات الإستهلاك**				
				**في اليوم**	**في الأسبوع**	**في الشهر**	**خلال فترة الحمل**	**أبدًا**
	كيف تتناولين الدجاج عادةً؟	☐ سفينة دجاج بدون جلدة مقليّة<br>☐ سفينة دجاج مع جلدة مقليّة<br>☐ فخذ دجاج بدون جلدة مقليّ<br>☐ فخذ دجاج مع جلدة مقليّ<br>☐ اسكالوب دجاج		☐ سفينة دجاج بدون جلدة مشويّة<br>☐ سفينة دجاج مع جلدة مشويّة<br>☐ فخذ دجاج بدون جلدة مشويّ<br>☐ فخذ دجاج مع جلدة مشويّ<br>☐ اسكالوب دجاج مع جبنة وجانبون				
59	ناغتز Nuggets	**6 قطع**						
60	لحم بقر (ستيك 6 قطع)	**90 غ**						
	نوع اللحمة	☐ مدهنة		☐ هبرة				
61	لحم غنم	**90 غ**						
	نوع اللحمة	☐ مدهنة		☐ هبرة				
62	بيض	**بيضة واحدة**						
		☐ البياض فقط		☐ الصفار فقط		☐ مقليّ		☐ مسلوق
63	تونة	**90 غ**						
	نوع التونة	☐ معلّبة بالماء				☐ معلّبة بالزيت		
64	سردين معلب	**3 قطع<br>90 غ**						
65	سمك (غير التونة) مقلي	**90 غ**						
66	سمك (غير التونة) مشوي	**90 غ**						
67	ثمارالبحر: بلح البحر(les)umo، أو القشريات<br>مثل سلطعون (rabec)، سوريمي، قريدس...	**30 غ**						
68	جامبون	**شرحتين<br>أو 30 غ**						
69	جامبون حبش	**شرحتين<br>أو 30 غ**						

70	شاركوتري (غيرالجامبون): مثل سلامي، مورتديلا...)	**شرحتين أو 30 غ**						
71	قصبة، قلوب وأكباد	**30 غ**						
72	شاورما (لحمة أو دجاج)	**90 غ**						
73	هوت دوج	**قطعة**						
74	برغر	**قطعة**						
	نوع البرغر	☐ لحمة (بقر)	☐ دجاج		☐ سمك		☐ غير ذلك:	
75	عصافير	**حبتين**						
	طريقة التحضير	☐ مقليّة			☐ مشويّة			
	**الطعام**	**الحصة**	**عدد الحصص**	**عدد مرّات الإستهلاك**				
				**في اليوم**	**في الأسبوع**	**في الشهر**	**خلال فترة الحمل**	**أبدًا**
76	ضفادع مقلية	**حبتين**						
77	قاورما	**معلقتين كبيرتين**						
78	نقانق	**2 – 3 قطع**						
79	بسترما	**شرحة**						
80	سجق	**قطعة**						
81	بايكون (Bacon)	**شرحة أو 30 غ**						
82	سوشي	**قطعة**						
G	**توابل، مكسّرات وقلوبات**							
83	مايونيز عادي	**ملعقة صغيرة**						
84	مايونيز دايت	**ملعقة كبيرة**						
85	كاتشب	**ملعقة كبيرة**						
86	صلصة الصويا	**ملعقة كبيرة**						
	نوع الصلصة	☐ عاديّة			☐ قليلة الملح			
87	مكعّب مرق	**مكعّب**						
88	كاجو، لوز	**6 حبّات**						
	النوع	☐ نيّ		☐ محمّص غير مملّح		☐ محمّص و مملّح		
89	فستق	**6 حبّة**						

	النوع	☐ نيّ	☐ محمّص غير مملّح	☐ محمّص و مملّح				
90	جوز	حبتين						
91	صنوبر	ملعقة كبيرة						
92	بندق	8 حبّات						
93	زبدة الفول السوداني	ملعقة كبيرة						
94	طحينة (بما في ذلك في الأطباق المطبوخة)	ملعقتين صغيرتين						
95	أفوكادو	شرحة						
96	زيتون	5 – 8 حبات						
97	جوز الهند	ملعقتين كبيرتين						
98	بزر اليقطين أو غيرها	ملعقة كبيرة						
	**الطعام**	**الحصة**	**عدد الحصص**	**عدد مرّات الإستهلاك**				
				**في اليوم**	**في الأسبوع**	**في الشهر**	**خلال فترة الحمل**	**أبدًا**
**H**	**السكاكر والحلويات**							
99	سكر، عسل، مربى، دبس، حبة بانبون	ملعقة كبيرة						
100	شوكولا المرح (مثل: نوتيلا)	ملعقة صغيرة						
101	شوكولا بالحليب، شوكولا مرّ	10 غ<br>أو مربع شوكولا						
102	شوكولا لوح	حسب الماركة						
	حددي الماركة:							
103	شوكولا بالوايفر	حسب الماركة						
	حددي الماركة:							
104	حلاوة	ملعقة كبيرة						
105	بسكويت دون كريمة	قطعة متوسطة						
106	بسكويت مع كريمة	قطعة متوسطة						
107	كيك ناشف	قطعة صغيرة						
108	كيك بالكريمة	قطعة صغيرة						
109	كريب، غوفر، بان كيك	30 غ<br>أو قطعة						

110	بوظة على حليب	**1 سكوب أو ½ كوب**						
111	بوظة على ثلج	**ستيك**						
112	بوظة سوربيه	**1 سكوب أو ½ كوب**						
113	بوظة على لبن	**1 سكوب أو ½ كوب**						
114	حلويات عربية (مثل: بقلاوة، معمول...)	**قطعة**						
115	حلويات عربية (مثل زنود الست، حلاوة الجبن...)	**قطعة**						
116	كنافة	**قطعة**						
117	أرز بالحليب، مهلبية، كاستر د	**كاسة**						
118	مغلي	**كاسة**						
119	جلو	**كاسة**						
		☐ عادي			☐ دايت			
120	اكلير، تارت بالفاكهة، ميل فاي	**قطعة**						
121	بيتي فور	**قطعة**						
	**الطعام**	**الحصة**	**عدد الحصص**	**عدد مرّات الإستهلاك**				
				**في اليوم**	**في الأسبوع**	**في الشهر**	**خلال فترة الحمل**	**أبدًا**
**I**	**المعجّنات**							
122	بيتزا	**قطعة**						
		☐ بوشيه			☐ مثلّثة			
123	مناقيش	**منقوشة حجم وسط**						
		☐ زعتر			☐ جبنة			☐ كشك
124	فطائر سبانخ	**قطعتين**						
125	فطائر جبنة و رقاقات	**قطعتين**						
126	لحم بالعجين	**قطعة**						
		☐ بوشيه			☐ حجم وسط			
127	كرواسان سادة، كرواسان زعتر	**قطعة**						
128	كرواسان جبنة	**قطعة**						
129	كرواسان شوكولا	**قطعة**						
130	خبز بالشوكولا، بان أوليه شوكولا	**قطعة**						

131	دونتس	**قطعة**						
132	بريوش	**قطعة صغيرة**						
**J**	**المأكولت المالحة**							
133	شيبس	**30 غ<br>أو 10 حبّات**						
134	بوشار (قليل الزيت)	**3 أكواب**						
135	بسكويت مملّح	**كيس**						
**K**	**صلصات، زيوت ودهون**							
136	زيت الزيتون	**ملعقة صغيرة**						
137	زيت دوّار الشمس	**ملعقة صغيرة**						
138	زيت الكانولا	**ملعقة صغيرة**						
139	زيت الذرة	**ملعقة صغيرة**						
140	زيت جوز الهند	**ملعقة صغيرة**						
141	زيت النخيل	**ملعقة صغيرة**						
142	زبدة عاديّة	**ملعقة صغيرة**						
143	سمنة نباتيّة	**ملعقة صغيرة**						
144	سمنة حيوانيّة	**ملعقة صغيرة**						

	**الطعام**	**الحصة**	**عدد الحصص**	**عدد مرّات الإستهلاك**				
				**في اليوم**	**في الأسبوع**	**في الشهر**	**خلال فترة الحمل**	**أبدًا**
**L**	**مشروبات**							
145	مياه معدنيّة	**كوب**						
146	شاي	**فنجان كبير**						
147	زهورات	**فنجان كبير**						
148	قهوة	**فنجان صغير**						
149	نسكافيه مع كوفي مايت	**فنجان كبير**						
150	نسكافيه دون كوفي مايت	**فنجان كبير**						
151	نسكافيه ديكاف مع كوفي مايت	**فنجان كبير**						
152	نسكافيه ديكاف دون كوفي مايت	**فنجان كبير**						

153	شاي مثلّج (ايس تي)	**كوب**						
154	مشروبات غازيّة (بيبسي..)	**كوب**						
155	مشروبات غازيّة دايت	**كوب**						
156	مشروبات الطاقة (ريد بل..)	**كوب**						
157	مشروبات روحيّة (كحول)	**كوب**						
