# Supplementary material for: Dietary patterns and birth outcomes of healthy Lebanese pregnant women
Source: Front Nutr. 2022 Sep 27;9:977288. doi: 10.3389/fnut.2022.977288 (PMC9551999; doi:10.3389/fnut.2022.977288)
Supplement: Supplementary file 2 [file Table_1.docx]

Supplementary Table 1: Food grouping used in factor analysis.

| **Food group** | **Food items** |
| --- | --- |
| Refined grains and cereals | White bread, Burger bun, Sandwich, Baguette, Pain au lait, Toast, Cracotte, Pain de mie, Kaak, Corn flakes, Cereal bar, Plain corn flakes |
| Whole grains and Legumes | Whole bread, Oat, Quinoa, Markouk, bulghur, Frike, Moghrabieh, Kibbeh, Fava beans, Beans, Lentils, Chickpeas |
| Rice and Pasta | Stuffed vegetables, Cooked pasta, |
| Starchy vegetables | Potato (Mashed and Pureed), Peas, Chestnut, Corn |
| Lebanese pies | Manaech, Ftayir, Lahm b ajjin |
| Dairy products | Milk, Laban, Labne, All types of cheese, Kechek |
| Fruits and Fruit Juices | All types of fruits, fresh fruit juices and sweetened fruit drinks |
| Vegetables (Raw and Cooked) | All types of vegetables |
| Eggs | All types of eggs |
| Fish | Grilled and fried fish, Tuna, Sardine, Seafood |
| Lean Meat | Poultry, Turkey |
| Medium to High Fat Meat | Steak, Organ meat, Processed meat, Bacon, Charcuteries, Lamb meat, Ham |
| Nuts and seeds | All types of nuts and seeds |
| Westernized Fast Food | Fries, Burger, Nuggets, Hot dog, Pizza, Ketchup, Soya sauce |
| Lebanese Fast Food | Shawarma, Awarma, Makanek, Basterma, Sujuk, |
| Sweets and Confectionery | Arabic sweets, Cakes, Cookies, Ice cream, Biscuits, Chocolate, Crepe, Pancakes, Honey, |
| Pastries | All types of croissants, Doughnuts, Brioche, Pain au chocolat, Brioche |
| Salty snacks | Chips, Popcorn, Salted biscuits |
| Monounsaturated Fat | Avocado, Tahini, Peanut butter, Olives, Olive oil, Mayonnaise diet |
| Polyunsaturated Fat | Canola oil, Sunflower oil, Corn oil, Mayonnaise |
| Saturated Fat | Coconut oil, Palm oil, Butter, All types of margarine |
| Sweetened Beverages | Soft drinks |
| Hot beverages | Coffee and Tea |

Supplementary Table 2: General characteristics of neonates.

| **Neonatal characteristics** | **Sexe** | **N** | **Mean** |  | **P Value** |
| --- | --- | --- | --- | --- | --- |
| **Height (cm)** | Female | 113 | 48.94 | 2.17 | 0.02^*^ |
|  | Male | 134 | 49.59 | 2.06 |  |
| **Weight (Kgs)** | Female | 113 | 3.05 | 0.48 | 0.01^*^ |
|  | Male | 134 | 3.20 | 0.44 |  |
| **Head circumference(cm)** | Female | 113 | 34.03 | 1.41 | 0.00^*^ |
|  | Male | 134 | 34.89 | 1.77 |  |
| **Apgar Score**  **( 1 min)** | Female | 113 | 8.71 | 1.26 | 0.73^*^ |
|  | Male | 134 | 8.76 | 1.01 |  |
| **Apgar Score**  **( 5 min)** | Female | 113 | 9.53 | 0.99 | 0.05^*^ |
|  | Male | 134 | 9.72 | 0.53 |  |

^*^ Statistical analyses done with Student *t* testwith a p value<0.05 considered as significant.
